# Cyanobacterial Diazotrophy and Earth’s Delayed Oxygenation

**DOI:** 10.3389/fmicb.2016.01526

**Published:** 2016-09-23

**Authors:** Stephanie L. Olson, Christopher T. Reinhard, Timothy W. Lyons

**Affiliations:** ^1^Department of Earth Sciences, University of California, Riverside, Riverside, CAUSA; ^2^School of Earth and Atmospheric Sciences, Georgia Institute of Technology, Atlanta, GAUSA

**Keywords:** cyanobacteria, oxygen, denitrification, diazotrophy, boring billion

## Abstract

The redox landscape of Earth’s ocean-atmosphere system has changed dramatically throughout Earth history. Although Earth’s protracted oxygenation is undoubtedly the consequence of cyanobacterial oxygenic photosynthesis, the relationship between biological O_2_ production and Earth’s redox evolution remains poorly understood. Existing models for Earth’s oxygenation cannot adequately explain the nearly 2.5 billion years delay between the origin of oxygenic photosynthesis and the oxygenation of the deep ocean, in large part owing to major deficiencies in our understanding of the coevolution of O_2_ and Earth’s key biogeochemical cycles (e.g., the N cycle). For example, although possible links between O_2_ and N scarcity have been previously explored, the consequences of N_2_ limitation for net biological O_2_ production have not been examined thoroughly. Here, we revisit the prevailing view that N_2_ fixation has always been able to keep pace with P supply and discuss the possibility that bioavailable N, rather than P, limited export production for extended periods of Earth’s history. Based on the observation that diazotrophy occurs at the expense of oxygenesis in the modern ocean, we suggest that an N-limited biosphere may be inherently less oxygenic than a P-limited biosphere—and that cyanobacterial diazotrophy was a primary control on the timing and tempo of Earth’s oxygenation by modulating net biogenic O_2_ fluxes. We further hypothesize that negative feedbacks inhibit the transition between N and P limitation, with the implication that the pervasive accumulation of O_2_ in Earth’s ocean-atmosphere system may not have been an inevitable consequence of oxygenic photosynthesis by marine cyanobacteria.

## Introduction

Cyanobacteria are responsible for two major metabolic innovations: (1) oxygenic photosynthesis and (2) aerobic N_2_ fixation. The former initiated the protracted oxygenation of Earth’s ocean-atmosphere system and eventually set the stage for the evolution of complex animal life (reviewed by Lyons et al., 2014). The latter is also a remarkable achievement considering that nitrogenase, the enzyme responsible for cleaving the dinitrogen triple bond, is irreversibly inactivated by O_2_ (reviewed by Berman-Frank et al., 2003). Despite the apparent incompatibility of oxygenesis and diazotrophy, cyanobacteria dominate N_2_ fixation in the modern well-oxygenated ocean (Capone et al., 1997). This uniquely cyanobacterial ability to reconcile both environmental (exogenous) and photosynthetic (endogenous) O_2_ with N_2_ fixation (diazotrophy) acts to minimize the potential for biospheric N limitation and favors primary production that is broadly limited by the bioavailability of P in the modern ocean (Redfield, 1958; Tyrrell, 1999). Nevertheless, the details of the co-evolution of cyanobacterial innovation, the O_2_ content of the ocean-atmosphere system, and Earth’s nutrient cycles during the Precambrian remain unclear. In particular, the origin of oxygenic photosynthesis and the oxygenation of Earth’s atmosphere appear to be temporally decoupled, and there are major uncertainties regarding the primary controls on the timing and tempo of Earth’s oxygenation (Lyons et al., 2014). The dynamics of the transition from a broadly anaerobic biosphere supported by NH_4_^+^ and anaerobic diazotrophy to an aerobic biosphere dominated by NO_3_^-^ assimilation and O_2_-tolerant cyanobacterial diazotrophy are also poorly known (Fennel et al., 2005), muddling our understanding of the causal relationships at play between O_2_ production, oxygenation, and marine nutrient cycling.

Herein, we examine the metabolic capabilities of extant diazotrophic cyanobacteria in the context of recent constraints on the oceanographic conditions of the mid-Proterozoic (~1.8 to 0.8 billion years ago or Ga). We discuss the possibility that the spatiotemporal redox landscape of the mid-Proterozoic ocean would have been less than ideal for cyanobacterial N_2_ fixation, favoring widespread N scarcity rather than P limitation. We also explore the implications of N limitation for biogenic O_2_ fluxes from the marine biosphere, hypothesizing that cyanobacterial production would have been less oxygenic under the O_2_ and nutrient conditions that typified the mid-Proterozoic ocean—potentially providing a biological mechanism for stabilizing *p*O_2_ at values much lower than those of the modern Earth.

## Earth’s Redox Evolution

The origin of cyanobacterial oxygenic photosynthesis significantly predates the permanent and pervasive oxygenation of Earth’s ocean-atmosphere system (Lyons et al., 2014). A growing collection of trace metal and isotopic records collectively indicates the accumulation of dissolved O_2_ in highly productive regions of the shallow ocean up to 50–500 million years before the initial oxygenation of the atmosphere ~2.4 Ga (e.g., Anbar et al., 2007; Kendall et al., 2010; Planavsky et al., 2014a)—a conclusion that is supported by recent biogeochemical models (Olson et al., 2013; Reinhard et al., 2013a). Evidence for dissolved O_2_ prior to the initial oxygenation of Earth’s atmosphere in the early Paleoproterozoic includes N isotope records that capture the onset of coupled nitrification and denitrification, implying metabolic utilization of O_2_ in the oxidation of NH_4_^+^ to N oxyanions in the surface ocean during the late Archean (Garvin et al., 2009; Godfrey and Falkowski, 2009). It is likely that dissolved O_2_ also accumulated, at least locally, in freshwater environments and within microbial mats, in isolation from prevailing oceanic and atmospheric conditions (Herman and Kump, 2005; Lalonde and Konhauser, 2015; Sumner et al., 2015). As such, inhibition of nitrogenase by O_2_ may have been an issue in both terrestrial and marine oxygen oases well before the first rise of atmospheric oxygen, and an initial inability to fix N_2_ aerobically to replenish bioavailable N lost through denitrification may have limited cyanobacterial proliferation in the late Archean (Scott et al., 2011).

It is also clear that Earth’s protracted oxygenation was not unidirectional and featured dramatic variability on a variety of timescales in addition to spatial heterogeneity (Lyons et al., 2014). Although relatively high atmospheric *p*O_2_ has been suggested during the Paleoproterozoic Lomagundi Event ~2.3–2.1 Ga (Bekker and Holland, 2012; Rybacki et al., 2016), several geochemical records collectively suggest that environmental O_2_ levels subsequently fell precipitously at the onset of the ensuing Boring Billion (~1.8–0.8 Ga) and remained relatively low until the late Neoproterozoic (Planavsky et al., 2012; Partin et al., 2013). Recent O_2_ constraints suggest mid-Proterozoic *p*O_2_ of less than 10^-3^ times the present atmospheric level as a long-term average (Planavsky et al., 2014b), with the implication that the dissolved O_2_ landscape of the mid-Proterozoic ocean was not markedly different than that of the late Archean (Reinhard et al., 2016). The emerging view is that the surface ocean was likely characterized by low-micromolar O_2_ that was highly variable in space and time (Reinhard et al., 2016), while the deep ocean remained anoxic (Poulton and Canfield, 2011)—for the vast majority of Earth history. Indeed, despite evidence for oxygenation in the late Neoproterozoic (Sahoo et al., 2012; Planavsky et al., 2014b), clear evidence for pervasive and permanent oxygenation within the ocean is lacking until the Phanerozoic (Kendall et al., 2015; Sperling et al., 2015). In this view, the so-called Great Oxidation Event of the early Paleoproterozoic has relatively minor ecological implications compared to subsequent oxygenation in the late Neoproterozoic and early Phanerozoic. Meanwhile, it remains unclear why biospheric O_2_ production failed to result in stably oxygenated surface environments and deep ocean oxygenation for ~2.5 billion years after cyanobacterial emergence.

## Oxygenation and Denitrification

The persistence of O_2_ stratification, that is, the widely held view that O_2_ was mostly restricted to the shallow ocean until the Paleozoic, has profound implications for the operation of the N cycle during Precambrian time. Denitrification, the reduction of bioavailable NO_3_^-^ to N_2_ via anaerobic respiration of organic matter, results in a substantial loss of bioavailable N in O_2_-deficient environments. Anaerobic oxidation of NH_4_^+^ coupled to NO_2_^-^ reduction (anammox) also represents a leak for bioavailable N as inert N_2_ (Kuypers et al., 2005). In the modern well-oxygenated ocean, denitrification and anammox are limited to organic-rich sediments and oxygen minimum zones (OMZs) underlying high-nutrient waters where the subsurface demand for respiratory electron acceptors locally exceeds O_2_ supply (Gruber and Sarmiento, 1997). Denitrification and anammox would also have been limited prior to the origin of oxygenic photosynthesis as the result of limited NO_2_^-^ and NO_3_^-^ synthesis in an anoxic ocean. The redox-stratified water column of the Proterozoic Earth, however, would have been particularly conducive to widespread loss of bioavailable N, because surface oxygenation would have allowed oxidation of NH_4_^+^ to N oxyanions by O_2_ in surface environments to support continued subsurface N_2_ synthesis via denitrification and anammox (Fennel et al., 2005; Garvin et al., 2009; Godfrey and Falkowski, 2009).

## Oxygenation and N_2_ Fixation

The likelihood of extensive loss of fixed N through denitrification requires a correspondingly large influx of bioavailable N via diazotrophy at steady-state; if N_2_ fixation cannot keep pace with denitrification, export production must decline until denitrification and N_2_ fixation are balanced (e.g., Deutsch et al., 2007). Thus, widespread denitrification may potentially modulate the long-term oxidation dynamics of Earth’s ocean-atmosphere system by limiting the burial of reduced carbon and sulfur if N_2_ fixation was less efficient than today. Indeed, there are several reasons that the Proterozoic surface ocean may have been less than ideal for cyanobacterial diazotrophy. Based on the metabolic capabilities of extant cyanobacteria, we suggest that the low-level, spatially and temporally variable O_2_ concentrations that likely characterized the Proterozoic surface ocean would have posed a worst-case scenario for N_2_ fixation by cyanobacteria—thus allowing for the possibility of an N-limited biosphere for much of Earth history.

Extant diazotrophic cyanobacteria differ dramatically in their ability to cope with environmental O_2_, and many N_2_ fixing cyanobacteria are actually obligate aerobes despite nitrogenase sensitivity to O_2_ (reviewed by Fay, 1992; Gallon, 1992). For these cyanobacteria, aerobic respiration provides: (1) a strategy for protecting nitrogenase by contributing to the maintenance of low intracellular O_2_ and (2) an energy source to support the demanding task of cleaving the dinitrogen triple bond while simultaneously maintaining O_2_ defense mechanisms. For example, despite the need for anoxic conditions *within* heterocysts, these differentiated cells are metabolically costly, and the rate at which O_2_ can diffuse into heterocysts can be an important control on the rate of N_2_ fixation in low O_2_ environments (Stal, 2009). Among modern heterocystous cyanobacteria, the metabolic machinery for N_2_ fixation appears to be optimized for present day air-saturated or even modestly elevated O_2_ concentrations (e.g., ~300 μM for *Anabaena cylindrica*; see Tomitani et al., 2006). Ambient O_2_ concentrations also appear to control both the rate and frequency of heterocyst growth (Kangatharalingam et al., 1992), with heterocysts grown under O_2_-replete conditions demonstrating lower sensitivity to environmental instability (e.g., diurnal fluctuations in O_2_ availability; Rippka and Stanier, 1978). In sum, the ability to cope with inhibitory levels of O_2_ via heterocystic cellular differentiation seems, rather ironically, to be favored by persistently elevated O_2_. These observations highlight the possibility that heterocystous diazotrophy may have been challenging for marine cyanobacteria for the vast majority of Earth history. Indeed, unambiguous marine heterocyst fossils are absent until the Phanerozoic (Golubic et al., 1995), and heterocystous cyanobacteria are relatively uncommon in well-oxygenated environments of the modern pelagic ocean (Zehr, 2011).

The non-heterocystous cyanobacteria that are capable of aerobic diazotrophy are generally tolerant of lower O_2_ concentrations than the heterocystous cyanobacteria. Nonetheless, some non-heterocystous cyanobacteria are also obligate aerobes, and low O_2_ availability may also reduce the rate of non-heterocystous N_2_ fixation (reviewed by Fay, 1992). Most aerobic non-heterocystous diazotrophic cyanobacteria optimally fix N_2_ at dissolved O_2_ concentrations on the order of ~10–50% of present marine levels (see Tomitani et al., 2006). Thus, it is likely that Proterozoic shallow marine dissolved O_2_ concentrations (~1% of modern; Reinhard et al., 2016) would have been suboptimal for diazotrophic cyanobacteria that were obligate aerobes. Meanwhile, even ~1% of modern dissolved O_2_ is sufficient to completely inhibit anaerobic N_2_ fixation among the majority of microaerobic non-heterocystous cyanobacteria (Bergman et al., 1997) and the obligate anaerobes capable of diazotrophy (e.g., some methanogens; Murray and Zinder, 1984).

## N Scarcity and Net O_2_ Production

Not all O_2_-tolerant, N_2_-fixing cyanobacteria meet the energetic demands of N_2_ fixation through aerobic respiration. Although O_2_ is not typically limiting in the open ocean today, one of the most successful marine diazotrophs, *Trichodesmium*, does not depend on exogenous O_2_ to respire stored carbohydrates. Instead, *Trichodesmium* is able to use its photosynthetic enzymes to power N_2_ fixation (reviewed by Bergman et al., 2013). However, because *Trichodesmium* performs diazotrophy at the expense of photosynthesis during daylight, midday O_2_ production is markedly reduced and potentially reversed when bioavailable N is limiting (Berman-Frank et al., 2001). Several other widespread unicellular marine cyanobacteria also fix N_2_ during the day using electron transfer from photosystem I (PSI)—and these cyanobacteria completely lack the ability to perform oxygenic photosynthesis (Zehr et al., 2008; Bothe et al., 2010; Tripp et al., 2010). These alternative strategies for coping with N scarcity would have been particularly advantageous if the ancient marine O_2_ landscape was not persistently favorable for either obligately aerobic or anaerobic N_2_ fixation. If PSI-dependent N_2_ fixation by O_2_ tolerant diazotrophic cyanobacteria was more common during Proterozoic time, cyanobacterial production may have been widely anoxygenic for much of Earth history. It follows that even if previous claims that the C isotope record precludes major changes in the burial of reduced organic carbon are correct (e.g., Holland, 2002), the C isotope record does not rule out major changes in net O_2_ fluxes from the marine biosphere through time.

This potential for reduced net oxygenesis in a low-O_2_ ocean is potentially exacerbated by the enhanced ecological significance of anoxygenic phototrophy coupled to the oxidation of H_2_S (Johnston et al., 2009; Hamilton et al., 2016). Indeed, biomarkers and several inorganic proxy records point to local subsurface H_2_S accumulation (euxinia) during the Proterozoic (Brocks et al., 2005; Scott et al., 2008; Lyons et al., 2009), despite the apparent persistence of iron-rich (ferruginous) conditions at depth (Planavsky et al., 2011; Poulton and Canfield, 2011). In addition to supporting greater anoxygenic production, euxinia may also increase the likelihood of nitrogenase metal cofactor limitation due to the burial of Mo in euxinic sediments (e.g., Anbar and Knoll, 2002)—even if euxinia was limited to only ~1–10% of the seafloor (Reinhard et al., 2013b). Regardless, because anaerobic respiration using SO_4_^2-^ as an electron acceptor (sulfate reduction) yields less energy than denitrification, evidence for euxinia is synonymous with evidence for NO_3_^-^ depletion (Boyle et al., 2013). The observation that euxinia was relatively common in both space and time during the Proterozoic is therefore fully consistent with the conclusion that N stress may have been problematic for the Proterozoic biosphere, as well as the assertion that the Proterozoic biosphere may have been less oxygenic.

## Oxygen-Nutrient Feedbacks

Whereas diazotrophy provides a pathway to alleviate N limitation, P is exclusively sourced to the ocean through crustal weathering. This inability of the marine biosphere to replenish P gives rise to the paradigm of a P-limited biosphere throughout Earth history (Redfield, 1958; Tyrrell, 1999). However, the assumption that cyanobacterial N_2_ fixation could keep pace with P supply during the Precambrian requires revisiting for reasons discussed above. In contrast with the prevailing view that P has limited marine productivity throughout Earth history, we hypothesize that prolonged N deficiency may have been a primary control on biospheric O_2_ fluxes and the tempo of Earth’s oxygenation.

**FIGURE 1:**
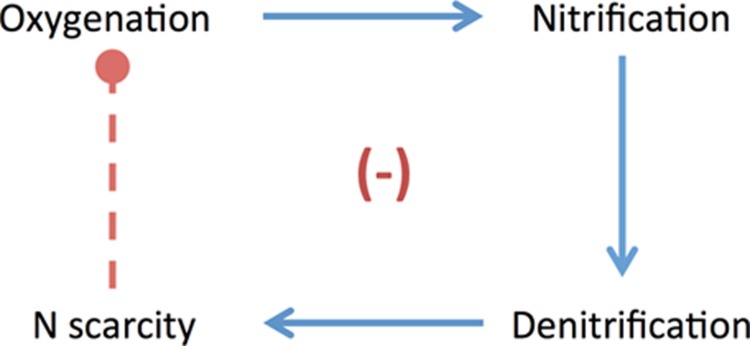
**Nutrient-O_2_ feedbacks.** Positive couplings are represented by solid blue arrows; negative couplings are represented by dashed red arrows. In the scenario shown here, minor surface oxygenation encourages nitrification, which allows greater subsurface denitrification (positive couplings). Loss of bioavailable N through denitrification in turn prompts greater N_2_ fixation—and we suggest that enhanced biospheric dependence on cyanobacterial diazotrophy results in reduced net O_2_ production (a negative coupling). The consequence is a stabilizing negative feedback: minor oxygenation results in reduced O_2_ production, strongly disfavoring O_2_ accumulation.

As long as the ocean remained stratified with respect to O_2_, subsurface anoxia would have allowed a positive coupling between surface oxygenation and denitrification: nitrification is enhanced as surface oxygen oases expand, allowing greater loss of fixed N through subsequent subsurface denitrification—promoting N scarcity and providing a stabilizing negative feedback on biogenic O_2_ accumulation (**Figure 1**; also see Fennel et al., 2005). Complete alleviation of N scarcity via N_2_ fixation would be challenging for the reasons outlined above, which may result in reduced export production and organic C burial in response to oxygenation. Meanwhile, we argue that enhanced biospheric dependence on diazotrophy may also result in a reduction of net O_2_ production per mol organic C burial. Thus, minor oxygenation of a redox-stratified biosphere may strongly disfavor further oxygenation through the reduction of net biological O_2_ production.

The critical coupling between oxygenation and denitrification is reversed under O_2_-replete conditions. Following the oxygenation of the deep ocean, increases in O_2_ would tend to limit the areal extent of denitrifying conditions in the ocean, minimizing the likelihood of N stress and favoring P limitation (Fennel et al., 2005). Meanwhile, net O_2_ production would be expected to increase as the biospheric dependence on N_2_ fixation and the contribution of anoxygenic phototrophy to export production decreased. Thus, there may be two stable oxygen-nutrient states for the Earth system following the origin of oxygenic photosynthesis: an O_2_-stratified, N-limited biosphere and an O_2_-replete, P limited biosphere (Anbar and Knoll, 2002; Fennel et al., 2005). Indeed, updated C isotope models suggest a permanent increase in organic burial at the end of the Proterozoic (Krissansen-Totton et al., 2015), possibly consistent with the alleviation of N stress and the transition to a P-limited biosphere as the consequence of oxygenation in the late Neoproterozoic.

## Concluding Remarks

Aerobic diazotrophy was a critical cyanobacterial innovation on par with oxygenic photosynthesis in terms of ecological impact, but it is likely that its environmental context and its contribution to global N cycling has varied dramatically through time. An important implication is that net biogenic O_2_ fluxes may have also varied through time. We hypothesize that low O_2_ availability may perpetuate low O_2_ conditions in the surface ocean, and anoxia at depth, by promoting extensive denitrification and the maintenance of conditions that are unfavorable for diazotrophy, both aerobic and anaerobic. Based on the success of anoxygenic diazotrophic cyanobacteria in the modern ocean, we further suggest that the most favorable cyanobacterial strategies for coping with N deficiency may have resulted in markedly reduced O_2_ production relative to today.

Enhanced biospheric dependence on cyanobacterial diazotrophy during the mid-Proterozoic, therefore, may explain the ~2.5 billion years delay between the Archean origin of oxygenic photosynthesis and the pervasive oxygenation of the ocean-atmosphere system in the Phanerozoic. Considering the likelihood of negative feedbacks that would tend to inhibit mitigation of global N-limitation, the relatively recent transition away from a world in which O_2_ accumulation was spatially limited to oases within the surface ocean toward a more pervasively oxygenated ocean may not be readily understood as the inevitable consequence of continued photosynthetic O_2_ production in surface environments. Instead, the transition from an O_2_-stratified, N-deficient marine biosphere to the broadly P-limited and well-oxygenated biosphere may require a yet unknown external perturbation to Earth’s O_2_ cycle. Although uncertainties remain, it is clear that cyanobacteria have played a highly dynamic role in the protracted oxygenation of the Earth system throughout the Precambrian—extending well beyond their invention of oxygenic photosynthesis roughly three or more billion years ago.

## Author Contributions

SO wrote the paper with insight from all authors.

## Conflict of Interest Statement

The authors declare that the research was conducted in the absence of any commercial or financial relationships that could be construed as a potential conflict of interest.
